# The role of autism and alexithymia traits in behavioral and neural indicators of self-concept and self-esteem in adolescence

**DOI:** 10.1177/13623613241232860

**Published:** 2024-02-27

**Authors:** Renske van der Cruijsen, Sander Begeer, Eveline A Crone

**Affiliations:** 1 Radboud University Nijmegen, The Netherlands; 2 Vrije Universiteit Amsterdam, The Netherlands; 3 Erasmus University Rotterdam, The Netherlands

**Keywords:** adolescents, alexithymia, autism spectrum disorder, functional magnetic resonance imaging, self-concept, self-esteem

## Abstract

Self-concept develops during adolescence, but little is known about self-concept in adolescents with autism. This behavioral neuroimaging study investigated (1) self-concept positivity across three domains (academic, physical appearance, and prosocial) and (2) from the perspective of self (direct self-concept) and the perceived perspective of peers (reflected self-concept) in 12- to 16-year-old adolescent males with (*n* = 35) and without autism (*n* = 34). These behavioral and neural measures of self-concept were additionally related to autism traits and alexithymia traits across groups. Results showed no general group differences, but more autism traits were related to less positive self-concept ratings in the physical appearance and prosocial domains. More autism traits were also associated with less similarity between direct and reflected prosocial self-concept ratings. Lower self-esteem was additionally explained by alexithymia, specifically the difficulty to identify ones feelings. Participants showed medial prefrontal cortex activation in response to evaluating self-traits in both groups. Region-of-interest analyses revealed that medial prefrontal cortex and right temporal–parietal junction activation were differentially related to alexithymia traits. Together, this study provides a comprehensive understanding of self-concept and self-esteem in adolescents with varying levels autism and alexithymia traits.

**Lay abstract:**

Developing a positive view of the self is important for maintaining a good mental health, as feeling negative about the self increases the risk of developing internalizing symptoms such as feelings of depression and anxiety. Even though autistic individuals regularly struggle with these internalizing feelings, and both self-concept and internalizing feelings are known to develop during adolescence, there is a lack of studies investigating the development of positive self-concept and self-esteem in autistic adolescents. Here, we studied academic, physical, and prosocial self-concept as well as self-esteem in adolescent males with and without autism on both the behavioral and neural level. We additionally focused on similarities in one’s own and peers’ perspectives on the self, and we assessed a potential role of alexithymia (i.e. having trouble identifying and describing one’s feelings) in developing a more negative view of the self. Results showed that there were no group differences in self-esteem, self-concept, or underlying neural activation. This shows that autistic adolescent males use the same neural processes when they evaluate their traits. However, regardless of clinical diagnosis, a higher number of autism traits was related to a less positive physical and prosocial self-concept, whereas more difficulty identifying one’s feelings was related to lowered self-esteem and less activation in medial prefrontal cortex during self-evaluations. Therefore, in treatment of autistic adolescents with low self-esteem, it is important to take into account and possibly aim to improve alexithymic traits as well.

## Introduction

An important task in adolescence is to develop a stable concept of self. Self-concept is defined as an estimation or evaluation of one’s own qualities or characteristics (Bailey, 2003). It has been proposed that the construction of one’s self-concept depends on cognitive abilities and social experiences: interactions with and feedback from others is important for developing a positive and accurate sense of self (Harter, 2012). Self-concept can therefore be evaluated from a direct personal perspective or from a reflected perspective, where the latter indicates the perceived opinions of others about the self (Harter, 2012; Jankowski et al., 2014; van der Cruijsen et al., 2018, 2019). Autistic individuals often experience difficulties in social situations and communication (American Psychiatric Association, 2013). They tend to have fewer and qualitatively different affective relationships and can experience difficulties in forming and maintaining close friendships (Fuentes et al., 2012; Kuo et al., 2013; Petrina et al., 2014). Nevertheless, there is still limited comprehension regarding self-concept in adolescents diagnosed with autism spectrum conditions (ASCs).

Several processes regarding self-concept are of interest during adolescence. First, adolescence may be a transition period for self-concept positivity and self-esteem, which are two closely related but different constructs (Crone et al., 2022). Specifically, self-esteem encompasses the overall evaluation of one’s worth or value as a person (Bailey, 2003; Harter, 2012) and is therefore an affective monitor of self-concept (Crone et al., 2022). In mid-adolescence, there is a relative dip in self-concept positivity (van der Cruijsen et al., 2018, 2023), and self-esteem is also reported to decline at this age (Robins & Trzesniewski, 2005), although this may depend on personal situations (Oshri et al., 2017). Even though some previous studies have shown that autistic adolescents (8–16 years) have lower self-esteem compared to age-matched non-autistic adolescents (Burrows et al., 2017; McCauley et al., 2019; Nguyen et al., 2020; Pfeifer et al., 2013; van der Cruijsen & Boyer, 2021), there have not been many studies investigating self-concept positivity among autistic adolescents. A prior study reported that 6- to 12-year-old children with autism, compared to their peers, used fewer positive statements to describe themselves (Begeer et al., 2008), but no difference was observed in positive self-descriptions in young adults aged 21–28 years with and without autism (Cygan et al., 2018). In this study, we intended to include both indicators of positivity about the self in order to facilitate a better understanding of these constructs in adolescents with autism.

Second, self-concept can differ between domains in adolescence (van der Cruijsen et al., 2018). Studies on self-competence (focusing on abilities rather than characteristics) showed that autistic adolescents rate social and athletic self-competence lower compared to non-autistic adolescents, whereas academic competence, physical appearance, and behavioral conduct ratings did not differ between groups (Bauminger et al., 2004; Vickerstaff et al., 2007; Williamson et al., 2008).

Third, it has been proposed that (perceived) opinions of others about the self are used to construct one’s self-concept (Harter, 2012; Van der Cruijsen et al., 2019). Individuals with ASC can have trouble with inferring others’ mental states (i.e. Theory of Mind (ToM); Begeer & Scheeren, 2021), and it has been proposed that these individuals may have a lower tendency to reason about others’ opinions of the self and to make reflected self-evaluations (Pfeifer et al., 2009; Pfeifer & Peake, 2012). Therefore, it would be informative to examine reflected versus direct self-concept in adolescents with autism.

Recent studies used neuroimaging methods to examine self-concept, given that self-concept is strongly dependent on self-report and neural correlates of self-concept evaluations may provide important additional insights. Task-related functional magnetic resonance imaging (fMRI) allows for the study of neural activity during the process of thinking about one’s own traits. Neuroimaging studies highlighted the medial prefrontal cortex (mPFC) as a key region involved in self-referential processing in typically developing children, adolescents, and adults (Denny et al., 2012; Pfeifer & Peake, 2012; van Buuren et al., 2020; Van der Cruijsen et al., 2019). There is conflicting evidence suggesting that autistic individuals may process self-relevant information differently from non-autistic individuals. That is, some studies reported lowered mPFC activation in individuals with autism or with lower autism symptom severity when evaluating self-traits (Kennedy & Courchesne, 2008; Kim et al., 2016), whereas other studies showed similar mPFC activation in adolescents with and without autism during self-evaluations (Burrows et al., 2016; Cygan et al., 2018).

A second brain region that has been linked to integrating perspectives of others in self-concept is the temporal–parietal junction (TPJ) (Schurz et al., 2014). Neuroimaging studies on self-concept in late adolescents showed that TPJ activation is stronger for reflected than direct self-evaluations (Pfeifer et al., 2009; Veroude et al., 2014). Autistic individuals, in contrast, typically show less involvement of TPJ activation in basic mentalizing and ToM tasks compared to individuals without autism (Kana et al., 2015; Murdaugh et al., 2014). Therefore, one might expect that involvement of TPJ in reflected self-evaluations is declined in autistic compared to non-autistic adolescents.

### Alexithymia

Alexithymia is a sub-clinical condition characterized by difficulties in recognizing and describing one’s own emotions (Sifneos, 1973). Whereas prevalence in the general population is around 10%–15%, alexithymia co-occurs in 50%–55% of people with ASC (Hill et al., 2004; Kinnaird et al., 2019; Milosavljevic et al., 2016). The alexithymia hypothesis (Cook et al., 2013) suggests that emotion-related difficulties in individuals with autism originate from alexithymia rather than representing a core feature of autism. This raises the question whether alexithymia rather than autism traits may play a role in self-concept positivity and self-esteem of autistic adolescents.

In addition, alexithymia traits have been related to decreased mentalizing and perspective-taking abilities (Moriguchi et al., 2006), which was also reflected in neuroimaging studies showing that alexithymia is related to reduced brain activation during empathizing (insula), mentalizing (mPFC), and emotion processing (precuneus) (Bird et al., 2010; Moriguchi et al., 2006; van der Velde et al., 2013). In addition, a structural MRI study in autistic adults has related alexithymia to reduced covariance in social-emotional (frontal-insular) but not social-cognitive (dorsal mPFC, TPJ) networks (Bernhardt et al., 2014).

The potential role of alexithymia in behavioral and neural measures of reflected self-evaluation has not yet been examined.

### Current study

The goal of this study was to provide a comprehensive investigation of self-concept in autistic adolescents by examining (1) self-concept across domains and (2) direct versus reflected self-concept, and using behavioral and neural measurements. Participants evaluated their traits in the academic, physical appearance, and prosocial domain from their own perspective and the reflected perspective of their peers, while undergoing MRI scans. Participants were adolescent males with a clinically established autism diagnosis and typically developing adolescent males aged 12–16 years. Since autistic traits are represented on a spectrum between individuals, we tested for group differences as well as for relationships with autism traits across all participants.

We expected (1a) that self-concept positivity, especially in the prosocial domain, and self-esteem would be lower in autistic compared to non-autistic adolescents, and would be negatively related to the number of autism traits (Bauminger et al., 2004; McCauley et al., 2019; van der Cruijsen & Boyer, 2021; Williamson et al., 2008). Regarding reflected versus direct self-concept, we expected (1b) higher similarity in non-autistic compared to autistic adolescents, or in participants with fewer autism traits (Pfeifer et al., 2009; Pfeifer & Peake, 2012). As alexithymia may explain emotion-related problems in autistic individuals (Cook et al., 2013), and it has been found to be related to decreased perspective-taking skills (Moriguchi et al., 2006), we exploratively tested (1c) whether alexithymia would explain lowered self-concept, lower self-esteem, or larger differences between direct and reflected self-concepts above autism traits.

On the neural level, we expected (2a) self-related mPFC activation in adolescents with and without autism (Burrows et al., 2016; Cygan et al., 2018). Previous studies were conflicted regarding potential differences in this activation between adolescents with and without autism. Therefore, here we tested exploratively for group differences and relationships with autism traits across both groups. Next, we expected (2b) that TPJ activation for reflected self-evaluations, and differentiation in TPJ activation for reflected versus direct self-evaluations would be stronger in non-autistic adolescents compared to adolescents with autism, or in participants with fewer autism traits (Kana et al., 2015; Kennedy & Courchesne, 2008; Lombardo et al., 2011; Murdaugh et al., 2014). Potentially, given the difficulties with social skills adolescents with autism often face, differences in neural activation between autistic and non-autistic adolescents mainly become apparent in the prosocial domain. Last, as alexithymia traits have been related to reduced neural activation for affective and mentalizing processes (Bird et al., 2010; Moriguchi et al., 2006; van der Velde et al., 2013), we tested (2c) whether alexithymia explained lowered mPFC and TPJ activation above autism traits.

## Methods

### Participants

Participants were 40 adolescent autistic males and 37 non-autistic peers aged between 12.1 and 16.9 years. In total, five participants with and three participants without autism were excluded due to not completing the MRI scans (*N*_Autism_ = 1), or excessive head movements during the MRI scans (*N*_Autism_ = 4, *N*_Non-Autism_ = 3), resulting in a final sample of 35 adolescent males with autism and 34 non-autistic peers (see Table 1). IQ was estimated using the two subtests “vocabulary” and “block design” of the Dutch Wechsler Intelligence Scale for Children (WISC-III-NL; Kort et al., 2005), which are known to correlate strongly to full-scale IQ (M = 100, SD = 15; Sattler, 2001). Estimated IQ scores ranged from 80 to 135 and did not differ between groups (*t*(67) = 1.18, *p* = 0.241). See Table 1 for information on ethnicity and gross annual income.

**Table 1.** Demographic information.

	Non-autistic participants			Autistic participants			
	Range	M	SD	Range	M	SD	*p* value
Age	12.1–16.9	14.4	1.4	12.5–16.9	14.8	1.0	0.21
IQ	80–135	111	12	80–132.5	107.5	12.6	0.24
AQ	2–22	8.4	5.0	3–22	12.9	4.7	<0.001^a^
DIF	7–17	10.5	2.5	7–17	11.8	2.9	0.03^a^
DDF	5–15	9.0	2.9	5–15	10.0	2.5	0.14
Motion during scan	0.05–0.26	0.099	0.048	0.05–0.18	0.080	0.027	0.046^a^
		*n*	%		*n*	%	
Medication use		0	100		15	42.9	
Methylphenidate					12	34.3	
Antipsychotics					4	11.4	
Other					5	14.3	
Comorbidities		0	100		20	57.1	
Dyslexia					9	25.7	
ADHD					8	22.9	
PTSD					2	5.7	
Sensory integration disorder					2	5.7	
Other					4	11.4	
Ethnicity							
The Netherlands		31	91.2		33	94.3	
Western Europe		0	0		1	2.9	
South Africa		1	2.9		1	2.9	
USA		1	2.9				
Southeast Asia		1	2.9				
Gross annual income							
<€31.000–		2	5.9		3	8.6	
€31.000–€76.000		18	53		16	45.7	
>€76.000–		13	38.2		10	28.6	
Declined to disclose		1	2.9		6	17.1	

IQ: estimated based on 2 WISC/WAIS-III subtests: block patterns and similarities; AQ: autism quotient questionnaire measuring autism traits; DIF: alexithymia difficulty identifying feelings; DDF: alexithymia difficulty describing feelings; SD: standard deviation; ADHD: attention-deficit/hyperactivity disorder; PTSD: post traumatic stress disorder.

a Independent samples *t* test indicates significant difference between groups.

Participants with ASC were recruited by sending an email to parents of boys aged 12 to 16 years, who were registered at the Netherlands Autism Register (NAR; https://nar.vu.nl/english/what-is-the-nar). A clinical *Diagnostic and Statistical Manual of Mental Disorders* (4th ed., text rev.; DSM-IV-TR; White, 2012) or *Diagnostic and Statistical Manual of Mental Disorders* (5th ed., DSM-5) diagnosis of autism spectrum disorder (ASD; American Psychiatric Association, 2013) was previously, independent of this study, determined in all participants by a psychiatrist or certified psychologist. Twenty autistic adolescents had co-occurring diagnoses, of whom six adolescents had two. Comorbid diagnoses were dyslexia (9), ADHD (8), post traumatic stress disorder (PTSD) (2), sensory integration disorder (2), Gilles de la Tourette (1), anxiety disorder (1), behavioral disorder not otherwise specified (1), and nonverbal learning disability (1). Fifteen adolescents used medication. Twelve participants used methylphenidate, of which one additionally used citalopram and four additionally used antipsychotics (aripiprazole, risperidone (2x), pipamperone). In addition, one participant only used dexamphetamine, one only used atomoxetine, and one only used aripiprazole. Participants who used medication were asked to take medication as usual before the lab visit to minimize possible influences of co-occurring problems such as attention problems on task performance.

Non-autistic participants were 12- to 16-year-old males (selected on matching age and gender) who participated in the first timepoint of a larger study (van der Cruijsen et al., 2018). None of the participants had any clinical diagnosis, or used medication, as was reported by parents over the phone during study inclusion, and self-reported by the adolescent in the questionnaire.

### Self-report measures

#### Autism traits

To measure the number of autism traits, participants completed the abridged version of the Autism Spectrum Quotient (AQ-short; Hoekstra et al., 2011). The questionnaire consists of 28 items which can be answered on a scale of 1 (definitely agree) to 4 (definitely disagree). Items were scored as either 0 or 1, with answer options 1 and 2 coded as “0” and answer options 3 and 4 coded as “1,” or vice versa for items that needed to be reverse-coded. The internal consistency was α = 0.74 for participants with autism, and α = 0.81 for non-autistic participants.

#### Alexithymia

To measure alexithymia, participants completed the Alexithymia Questionnaire for Children (AQC; Rieffe et al., 2006), consisting of 20 items on which participants could respond on a scale of 1 to 3 (1 =not true, 2 = a bit true, 3 = true). This questionnaire consists of three subscales: difficulty identifying feelings (DIF; e.g. “I am often confused about what emotion I am feeling”), difficulty describing feelings (DDF; e.g. “It is difficult for me to find the right words for my feelings”), and externally oriented thinking (EOT; e.g. “I prefer to just let things happen rather than to understand why they turned out that way”). The internal consistency of the subscales was DIF: α = 0.74, DDF: α = 0.72, EOT: α = 0.34 for participants with autism, and DIF: α = 0.61, DDF: α = 0.80, EOT: α = 0.62 for participants without autism. Given the low reliability of the EOT subscale, we excluded the EOT subscale from further analyses.

#### Self-esteem

To measure adolescents’ self-esteem, participants completed the Dutch version of the Rosenberg Self-Esteem Scale (RSES), consisting of 10 items on which participants could respond on a scale of 1 (completely not true) to 4 (completely true) (Veldhuis et al., 2014). Internal consistency was α = 0.89 for participants with autism and α = 0.73 for participants without autism.

### Task design

#### FMRI self-concept task

**Figure 1.**
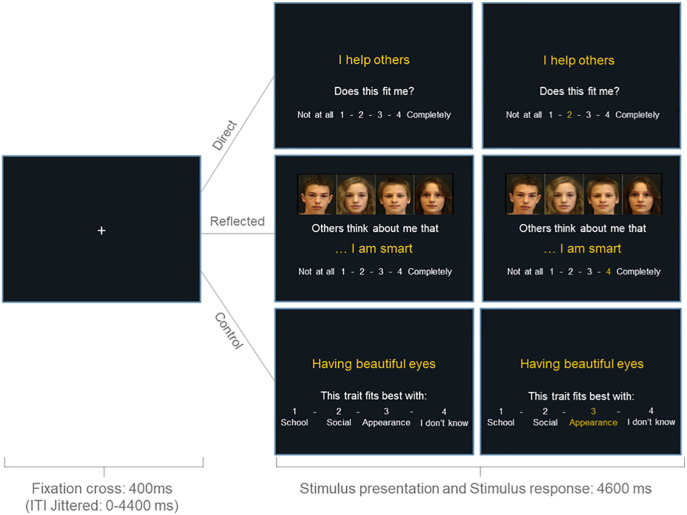
Example of a trial in the direct, reflected, and the control condition. Each trial started with a black screen and a jittered duration between 0 and 4400 ms. Subsequently, a fixation cross was shown for 400 ms after which the stimulus appeared. In the direct and reflected conditions, participants rated on a scale of 1 to 4 to what extent the traits described themselves (from their own perspective or their perceived peers’ perspective, respectively). In the control condition, participants categorized the trait sentences into one of four options. The stimulus was shown for 4600 ms. If participants responded within this timeframe, the number of their choice would turn yellow. If participants failed to respond within this timeframe, a screen with the phrase “Too Late!” was shown for an additional 1000 ms after which the next trial would start.

Participants evaluated the extent to which sentences describing positive and negative traits in academic, physical, and prosocial domains fit them on a scale of 1 (not at all) to 4 (completely) (see Figure 1). The task consisted of two experimental conditions and one control condition. In the experimental conditions, participants evaluated their traits from their own (direct self-evaluation condition) or their peers’ perspective (reflected self-evaluation condition). Trait sentences were the same in both conditions (i.e. “I am smart,” “I am unattractive”), but in the reflected condition were preceded by the words: “My peers think about me that …” Participants evaluated 60 trait sentences in both conditions: 20 sentences per domain, of which half were positive and half were negative. In the control condition, participants categorized 10 positive and 10 negative trait sentences different to those in the experimental conditions into one of four categories: (1) school, (2) social, (3) appearance, and (4) I don’t know. For behavioral analyses of self-concept positivity, responses to negative items were reverse-scored and averaged together with responses on positive items.

The three conditions were completed in separate runs of which the order was counterbalanced between participants. Within the runs, trials were presented in a pseudorandomized order regarding domains, optimized using Optseq (Dale, 1999). Optseq was also used to add jittered intertrial intervals, which varied between 0 and 4.4 s. Each trial began with a fixation cross shown for 400 ms, after which the stimulus was presented for 4600 ms. When participants successfully responded to the sentence within this timeframe, the number they chose turned yellow for the remaining stimulus time in order to assure participants that their choice had been registered. If participants failed to respond, they were shown the phrase “Too late!” for 1000 ms. These trials were modeled separately and were not included in the analyses. Too late responses for adolescents with autism and typically developing adolescents, respectively, occurred on 1.5% and 1.4% of trials in the direct condition, on 2.7% and 2.4% of trials in the reflected condition, and on 1.1% and 0.9% of trials in the control condition. Differences in missed responses were not significant between groups (all *p* > 0.687).

### FMRI preprocessing

Data were analyzed using SPM8 (Wellcome Department of Cognitive Neurology, London) for comparison with previously published studies (Van der Cruijsen et al., 2019). Functional scans were corrected for slice-timing acquisition and rigid body movement differences. Structural and functional volumes were spatially normalized to T1 templates by an algorithm using a 12-parameter affine transformation together with a nonlinear transformation involving cosine basis functions, resampling the volumes to 3-mm cubic voxels. Templates were based on the MNI305 stereotaxic space (Cocosco et al., 1996). Functional volumes were spatially smoothed with a 6-mm full width at half maximum (FWHM) isotropic Gaussian kernel.

Task effects for each participant were estimated using the general linear model (GLM) in SPM8. The fMRI data were modeled as a series of zero duration events convolved with the hemodynamic response function (HRF). Modeled events of interest for the direct condition were “Direct-Academic-Positive,” “Direct-Academic-Negative,” “Direct-Physical-Positive,” “Direct-Physical-Negative,” “Direct-Prosocial-Positive,” and “Direct-Prosocial-Negative.” The same events were modeled for the reflected condition. For the control condition, only one event of interest was modeled: “Control” (collapsed across domains and valences). Trials for which participants failed to respond in time were modeled as events of no interest. The events were used as covariates in a GLM, together with a basic set of cosine functions that high-pass filtered the data. Six motion regressors were added to the model. Participants who moved more than 3 mm in any direction were excluded from the analyses (*n* = 4 autistic adolescents and *n* = 3 non-autistic adolescents). The resulting contrast images, computed on a subject-by-subject basis, were submitted to group analyses.

For motion differences between groups, see Table 1. Both across groups and within both groups separately, motion was not related to autism traits, alexithymia traits, or self-esteem (all *p* > 0.063). Controlling for motion in all analyses did not change the results.

### FMRI whole-brain analyses

See supplement for fMRI data acquisition. To investigate our hypotheses, we first performed whole-brain one-sample *t* tests for the contrast Self (Direct + Reflected) > Control, separately for both groups. Subsequently, we performed a whole-brain two-sample *t* test for the difference in this same contrast between the groups. Family-wise error (FWE) cluster correction was applied in these analyses. To further investigate our hypotheses regarding mPFC and TPJ activation, we extracted parameter estimates from 3 regions of interest (ROIs; 8-mm spheres) using the MarsBaR ROI toolbox: mPFC (*x* = −6, *y* = 50, *z* = 4), right TPJ (x = −53, *y* = −59, *z* = 20), and left TPJ (*x* = 56, *y* = −56, *z* = 18). These ROIs were based on previous meta-analyses (Denny et al., 2012; Schurz et al., 2014) and have been used in our early study on self-concept development in adolescence (Van der Cruijsen et al., 2019).

### Behavioral and ROI analyses

Repeated-measures analyses of variance (ANOVAs) were conducted to examine group differences in behavior and neural activation in the three ROIs. Next, hierarchical regression analyses were conducted for two purposes. First, with these analyses we examined whether behavior and neural activation related to autism traits regardless of diagnosis. Second, by adding alexithymia traits in the next step of the regression, we tested whether alexithymia would explain additional variance in self-concept and self-related neural activation above autism traits.

Results were corrected for multiple comparisons using a Bonferroni method adjusting for correlated variables (http://www.quantitativeskills.com/sisa/calculations/bonfer.htm; Perneger, 1998; Sankoh et al., 1997). For the hierarchical regressions including behavioral measures, the correlation between the seven outcome variables was *r* = 0.314, which resulted in an adjusted significance level (two-sided) of α = 0.013. For the hierarchical regressions including the 12 ROI measures in the contrasts Self > Control, the average correlation was *r* = 0.503, resulting in an adjusted significance level of α = 0.0145. Last, for the hierarchical regressions including the 12 ROI measures in the contrasts Reflected > Direct, the average correlation was *r* = 0.4659, resulting in an adjusted significance level of α = 0.013. Even though all hierarchical regression analyses answered one overarching question (whether alexithymia rather than autism traits were related to indicators of self-concept and self-esteem), there were three variables of interest in these analyses. Therefore, we have reported when results would not survive an additional Bonferroni correction (α = 0.013/3 = α = 0.0043; α = 0.0145/3 = α = 0.0048; and α = 0.013/3 = α = 0.0043, respectively).

### Community involvement statement

Community members were not actively involved in the construction of this study. However, every year, the NAR exchanges ideas on relevant research topics with stakeholders such as autistic adults and parents of children with autism. The NAR also has several autistic team members.

## Results

### Behavioral results

First, we examined group differences in self-concept positivity (perspective, domain), self-esteem, and perspective similarity (reflected–directed). Second, we tested for relationships of these behavioral indicators with autism and alexithymia traits using hierarchical regression analyses.

#### Group analyses

##### Self-concept positivity

To test for differences in self-concept positivity between adolescents with and without autism, we conducted a Perspective (2: Direct/Reflected) × Domain (3: Academic/Physical Appearance/Prosocial) rmANOVA with “Group” as between-subjects factor and self-concept positivity as dependent measure. Medication use, IQ, and age were added as covariates of no interest. There were no main effects of Perspective, Domain, or Group, nor were there interaction effects between these variables (all *p*s > 0.091), indicating that self-concept positivity was similar in all domains for males with and without autism.

##### Self-esteem

A univariate ANOVA controlling for medication use, IQ, and age revealed no group differences in self-esteem (*F*(1,64) = 0.194, *p* = 0.661, η_p_^2^ = 0.003).

##### Perspective similarity

An additional way to examine the effect of Perspective is by calculating similarity. To measure similarity between direct and reflected self-evaluations, we calculated individual item-by-item correlations for matching items in the direct and reflected conditions. A Domain (3) rmANOVA for item–item agreement with “Group” as between-subjects factor and controlling for medication use, IQ, and age resulted in no main effects of Domain or Group and no Group × Domain interaction (all *p*s > 0.159).

#### Trait analyses

See Supplementary Table 1 for test statistics of all hierarchical regression analyses on behavioral measures.

##### Self-concept positivity

Next, we performed hierarchical regression analyses across all participants for self-concept positivity separately for each domain (three separate analyses) controlling for medication use, IQ, and age in step 1, including autism traits as a predictor in step 2, and alexithymia subscales DIF and DDF in the third step.

**Figure 2.**
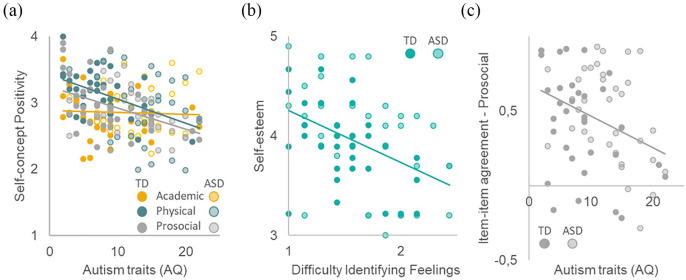
(a) Physical and prosocial self-concept positivity are negatively related to the number of autism traits. (b) Self-esteem is negatively related to the level of alexithymia difficulty identifying feelings. (c) More autism traits are related to lower item-by-item agreement between matching items in the direct and reflected self conditions in the prosocial domain.

Results showed that autism traits were negatively related to physical appearance self-concept positivity (*β* = −0.392, *t*(64) = −3.35, *p* = 0.001) and prosocial self-concept positivity (*β* = −0.459, *t*(64) = −3.89, *p* < 0.001). No significant relationship was found with academic self-positivity (*β* = −0.059, *t*(64) = −0.45, *p* = 0.654) (Figure 2(a)). Adding alexithymia subscales in the third step of the regression did not improve the three models for domain-specific self-concept positivity.

##### Self-esteem

The same analyses were performed with self-esteem as dependent measure. Results showed that autism traits were positively related to self-esteem (*β* = −0.343, *t*(64) = −2.74, *p* = 0.008; this did not survive second/additional Bonferroni correction). The model improved after adding alexithymia (Figure 2(b)). In this model, DIF was negatively related to self-esteem (*β* = −0.495, *t*(62) = −3.58, *p* < 0.001).

##### Perspective similarity

The same analyses were performed with similarity as dependent measure. The model for similarity showed that item–item agreement in the prosocial domain was negatively related to autism traits (*β* = −0.399, *t*(64) = −3.25, *p* = 0.002; Figure 2(c)). Adding alexithymia in the third step of the regressions did not improve model fits (*p* > 0.122). No effects were observed for the other domains (*p* > 0.098).

### Neural results

First, whole-brain contrasts for Self > Control conditions for both groups (autistic and non-autistic) were constructed. Next, ROI analyses examined the main hypotheses concerning neural activation for self-concept across domains and for perspective similarity. These analyses were first performed to compare groups using repeated-measures analyses. Second, we tested for relationships of neural activation with autism and alexithymia traits using hierarchical regression analyses.

#### Group level: self-related brain activation

##### Whole-brain contrasts

**Figure 3.**
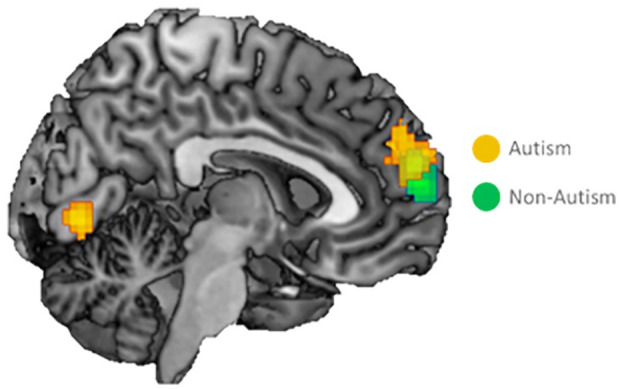
Whole-brain activation for the contrast Direct & Reflected Self > Control. Overlapping activation in medial prefrontal cortex for evaluating self-traits versus the control condition in adolescents with and without autism. Activation was corrected using family-wise error (FWE) cluster-level correction at *p* < 0.05, at an initial uncorrected threshold of *p* < 0.001.

The whole-brain contrasts Self (Direct + Reflected) > Control revealed similar activation in mPFC in both groups (Figure 3; Table 2). Autistic adolescents additionally activated right lateral PFC and right lingual gyrus, whereas non-autistic adolescents additionally activated inferior parietal/supramarginal gyrus. A whole-brain two-sample *t* test for Self > Control to test for significant differences between the groups did not result in significant effects, suggesting that self-related brain activation at a whole brain level was similar for adolescents with and without autism. Repeating analyses controlling for reaction times did not change the results.

**Table 2.** Regions activated for the contrast direct and reflected Self > Control.

Region	*BA*	Coordinates			Cluster size	*T*
ASD group						
R inferior frontal	44	54	11	16	62	5.70
R superior medial frontal	10	6	59	22	201	5.20
(mPFC)		−6	50	28		4.75
		−12	56	16		4.73
R superior frontal	6	18	5	58	74	5.03
R lingual	18	12	−79	−2	76	4.98
		3	−76	−2		4.93
TD group						
R superior medial frontal	10	9	56	13	136	5.20
(mPFC)		−3	56	22		4.52
		−9	56	7		4.31
R inferior parietal	40	57	−40	49	145	4.82
Supramarginal		57	−40	37		4.44

*Note.* Names were based on the Automatic Anatomical Labeling (AAL) atlas. FWEc for ASD group = 62, FWEc for TD group = 136. ASD: autism spectrum disorder; mPFC: medial prefrontal cortex; FEW: family-wise error; BAB: Brodmann area; TD: typically developing.

##### ROI analyses

An ROI analysis is meaningful to test for activation differences in pre-defined ROIs with more power. Therefore, we extracted parameter estimates from a pre-defined mPFC, left TPJ, and right TPJ ROI, and calculated activation per domain for direct self-evaluations versus the control task, and reflected self-evaluations versus the control task. We performed three Perspective (2) × Domain (3) rmANOVAs with Group as between-subjects factor and medication use, IQ, and age as covariates of no interest.

There were no differences in mPFC and left TPJ activation between Perspectives (mPFC: *p* = 0.420; left TPJ: *p* = 0.833), Domains (mPFC: *p* = 0.378; left TPJ: *p* = 0.873), or Groups (mPFC: *p* = 0.140; left TPJ: *p* = 0.652), and there were no interaction effects (mPFC: *p* > 0.167; left TPJ: *p* > 0.075).

For right TPJ, results showed a no main effects of Perspective (*p* = 0.223), Domain (*p* = 0.757), or Group (*p* = 0.460). There was a Perspective × Domain × Group interaction effect, but it did not survive Bonferroni correction (*F*(2,128) = 3.66, *p* = 0.029, η_p_^2^ = 0.054).

#### Trait level: self-related brain activation

##### ROI analyses

Next, we performed hierarchical regression analyses across all participants for all three ROIs in the Self > Control contrast and separately for each domain (12 separate analyses). In these analyses, we controlled for medication use, IQ, and age in step 1; included autism traits as a predictor in step 2; and added alexithymia subscales DIF and DDF in the third step. See Supplementary Table 2 for test statistics of all hierarchical regression analyses on the Self > Control ROI activation.

**Figure 4.**
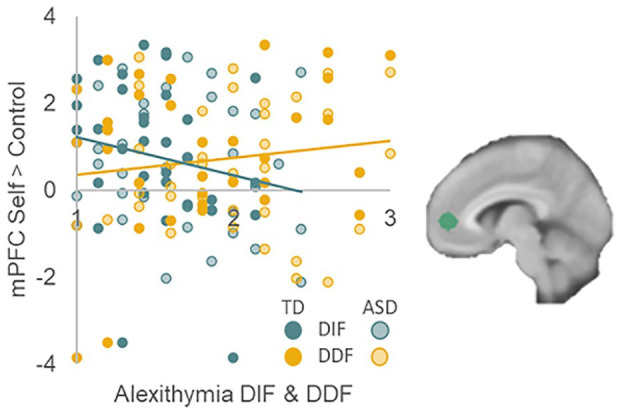
mPFC activation in response to self-evaluations is negatively related to alexithymia “difficulty identifying feelings” and positively related to alexithymia “difficulty describing feelings.” This was the case for mPFC activation across all domains, and for all domains individually. Only for mPFC activation in the physical domain, the relationship with DDF did not survive Bonferroni correction.

First, regression analyses showed that autism traits were not associated to self-related mPFC activation, neither across domains nor in the academic, physical, and prosocial domains separately (*p* > 0.600). Adding alexithymia in the third step of the regression improved model fit in all cases (all *p*_change_ < 0.017; Supplementary Table 2). DIF showed a negative relationship with self-related mPFC activation (all *p* < 0.007; did not survive second/additional Bonferroni correction for academic and physical domains), and DDF showed a positive relationship with self-related mPFC activation (all *p* < 0.010; both across domains and for all domains separately). The relationship between mPFC activation in the academic and physical domains and DDF did not survive Bonferroni correction (Figure 4; Supplementary Table 2).

Second, regression analyses showed that autism traits were positively associated to self-related left TPJ activation for the contrast Self > Control for the academic domain specifically (*β* = 0.303, *t*(64) = 2.37, *p* = 0.021; other *p* > 0.060), but this relationship did not survive Bonferroni correction. The addition of alexithymia in step 3 did not improve the models (*p* > 0.446).

Autism traits were not related to right TPJ activation (*p* > 0.377), nor did the addition of alexithymia in step 3 improve the models (*p* > 0.658).

#### Trait level: perspective similarity brain activation

##### ROI analyses

Next, we performed hierarchical regression analyses across all participants for a different contrast in the same ROIs. Here, we examined reflected-direct similarity separately for each domain. In these analyses, we controlled for medication use, IQ, and age in step 1; included autism traits as a predictor in step 2; and added alexithymia subscales DIF and DDF in the third step. See Supplementary Table 3 for test statistics of all hierarchical regression analyses on the Reflected > Direct ROI activation. Group differences were not examined using these rmANOVAs since Group × Perspective differences were already covered in the rmANOVAs on ROI data described above.

Regression analyses for mPFC indicated that autism traits were not related to the difference between reflected and direct self-evaluations, neither across domains nor in any of the domains separately (all *p* > 0.187). Adding alexithymia in the third step of the regression did not improve model fits (all *p*s > 0.070).

Regression analyses for left TPJ showed a negative relationship between autism traits and the difference between reflected and direct self-evaluations in the prosocial domain (*β* = −0.283, *t*(64) = −2.20, *p* = 0.032), although this relationship did not survive Bonferroni correction. Other relationships, and the addition of alexithymia in step three of the regression, were not significant (all *p*s > 0.081).

**Figure 5.**
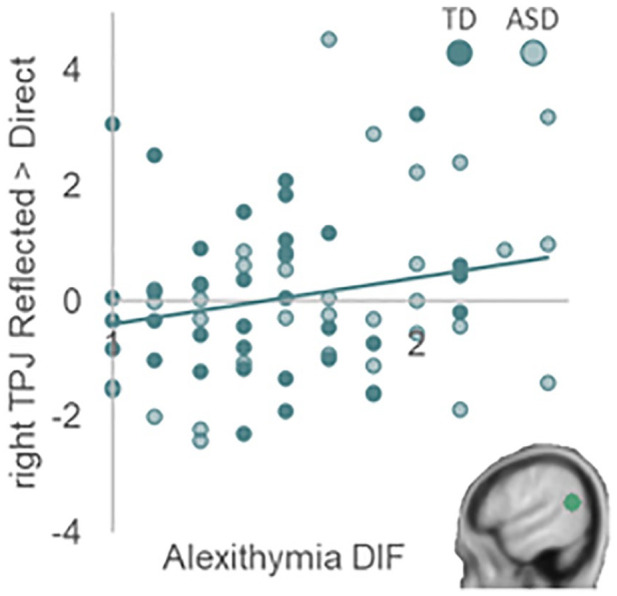
Right TPJ activation in response to reflected versus direct self-evaluations is positively related to alexithymia difficulty identifying feelings. This was the case for right TPJ activation across all domains and for the academic and physical appearance domains individually.

Regression analyses for right TPJ showed that autism traits were not related to neural activation for the difference between reflected and direct self-evaluations (all *p*s > 0.089). However, adding alexithymia DIF and DDF in step 3 of the regression significantly improved the model across all domains, and for the academic and physical domains specifically (across domains: *F*_change_(2, 62) = 4.79, *p*_change_ = 0.012; academic: *F*_change_(2, 62) = 5.00, *p*_change_ = 0.010; physical: *F*_change_(2, 62) = 4.38, *p*_change_ = 0.017). Here, DIF positively related to the difference in right TPJ activation between reflected and direct self-evaluations (across domains: *β* = 0.451, *t*(62) = 3.09, *p* = 0.003; academic: *β* = 0.463, *t*(62) = 3.09, *p* = 0.003; physical: *β* = 0.389, *t*(62) = 2.64, *p* = 0.010; did not survive second/additional Bonferroni correction for physical domain; see Figure 5). Alexithymia did not improve the model for the prosocial domain (*p* = 0.111).

## Discussion

This study examined self-concept in autistic adolescents in an experimental design that allowed us to dissociate self-concept positivity and self-concept perspective similarity. An additional aim was to examine predictive values of autism and alexithymia traits. Even though we did not observe differences in self-concept positivity when comparing adolescents with and without autism, autism traits across all participants were related to lower self-concept positivity (except for academic traits) and lower self-esteem. Alexithymia explained additional variance in self-esteem above autism traits. Self-related mPFC activation was observed for adolescents with and without autism, but was related to alexithymia traits. Next, item-level similarity for reflected and direct prosocial self-concept ratings showed higher similarity in individuals with fewer autism traits, and right TPJ activation was stronger for reflected versus direct traits in individuals with more alexithymia traits.

### Self-concept positivity and differences between domains

There was no evidence for a significant difference in self-concept positivity between adolescents with and without autism. We confirmed, however, the hypothesis of lower self-concept positivity in adolescents with more autism traits, across groups (Bauminger et al., 2004; McCauley et al., 2019; van der Cruijsen & Boyer, 2021; Williamson et al., 2008). We expected that this effect would be specific for the prosocial domain, but the negative relation was also observed for physical appearance traits. In contrast, no relation between autism traits and self-concept positivity was observed for academic traits, consistent with prior studies showing that academic competence is similar between groups with and without autism (Bauminger et al., 2004; Vickerstaff et al., 2007; Williamson et al., 2008).

There was no evidence for a difference in self-esteem in adolescents with and without autism. However, this study suggests that adolescents with more autism traits, across groups, may have lower general self-esteem, consistent with prior studies (McCauley et al., 2019; van der Cruijsen & Boyer, 2021). Interestingly, alexithymia (specifically DIF) explained additional variance in lower self-esteem (but not self-concept) above autism traits. This was not the case for self-concept which despite the inherently evaluative component can be seen as a relatively cognitive construct, whereas self-esteem is a more affective or emotional indicator of positivity about the self (Crone et al., 2022). This adds to the alexithymia theory such that in addition to previously studied emotional difficulties in autistic individuals, alexithymia plays a role in adolescents’ feelings of self-worth. Prior research showed that self-esteem is related to internalizing problems that also have been related to alexithymia, such as depression and anxiety (Bloch et al., 2021; McCauley et al., 2019; Milosavljevic et al., 2016; van der Cruijsen & Boyer, 2021). Therefore, self-esteem may be an important link in the development of adolescents’ mental well-being.

Autistic and non-autistic adolescents activated the same brain regions when evaluating self-traits, specifically the mPFC. This is consistent with recent studies in adolescents and early adults showing that autistic adolescents recruit similar underlying neural networks (Burrows et al., 2016; Cygan et al., 2018). Activation in mPFC for self-evaluations was negatively and positively related to different components of alexithymia. First, self-related mPFC activation was less pronounced for individuals with more DIF. Activation in mPFC underlying evaluation of self-traits proposedly reflects self-relevance or personal value (D’Argembeau, 2013; van der Cruijsen et al., 2017) and activation has been related to self-evaluations of positive compared to negative traits (D’Argembeau et al., 2008; van der Cruijsen et al., 2017, 2018). Having trouble identifying one’s feelings about the self may make the evaluation of one’s traits feel relatively less relevant or of less value to the self, associated with attenuated mPFC activation for self-evaluations. Second, DDF was positively related to mPFC activation, such that activation was stronger for individuals with more DDF. Even though DIF and DDF are correlated within individuals (Loas et al., 2001), this study highlights the importance of separately evaluating these components of alexithymia since they are differentially related to self-esteem and neural activation underlying self-evaluations. Future studies may aim to replicate and further investigate these distinct relationships of alexithymia components DIF and DDF with self-concept and related neural activation.

### Direct versus reflected self-concept

A second goal of this study was to investigate perspective similarity in self-evaluation. It has been theorized that the perceived opinions of others, especially peers, about the self (reflected self-concept), become internalized into one’s own opinion about the self (direct self-concept) (Dudovitz et al., 2017; Harter, 2012). This study showed that autistic and non-autistic adolescents were equally positive about themselves from the direct and reflected perspective. In addition, within-person similarity between direct and reflected self-evaluations did not differ between groups (Cage et al., 2016). However, similarity in the prosocial domain was related to autism traits across groups, such that self-evaluations of the same prosocial traits from the direct and perceived peers’ perspective were less aligned in adolescents with more autism traits (Hobson et al., 2006; Lee & Hobson, 1998). Possibly, adolescents with more autism traits are explicitly aware that their (pro)social traits are generally not well appreciated or understood by others (Carrington et al., 2003). Alternatively, adolescents with more autism traits may have more trouble estimating the opinions of others about their prosocial traits. Indeed, adolescents with autism generally struggle more to understand others’ thoughts and intentions, despite being able to form (ToM; Begeer & Scheeren, 2021).

This pattern was further investigated using neural ROI analyses. Neural activation in the TPJ for taking others’ versus own perspectives in self-evaluations did not differ between groups and was not related to autism traits across groups. This contradicts previous studies showing attenuated TPJ activation in individuals with ASC in processes involving ToM in different paradigms (Kana et al., 2015; Murdaugh et al., 2014). A prior study also showed more selective involvement of TPJ in mentalizing in individuals without autism (Lombardo et al., 2011).

There was, however, a relationship between right TPJ activation and alexithymia. That is, right TPJ activation was higher for reflected compared to direct self-evaluations in individuals with more DIF across domains and for the academic domain specifically. This does not directly align with previous studies that linked alexithymia to *attenuated* neural activation in regions involved in self-processing and mentalizing (Bird et al., 2010; Moriguchi et al., 2006; Van der Velde et al., 2013). Possibly, larger differentiation in TPJ activation between perspectives reflects either increased neural effort in aiming to reason from others’ perspectives or relatively lowered mentalizing activation during direct self-evaluations. Future studies may aim to replicate and break down this pattern.

### Limitations and future directions

This study had several limitations that should be addressed in future studies. First, it is noticeable that, although the design of this study was optimized to test for group differences, the results were mainly associated with severity of autism and alexithymia traits. Future studies should optimize their design to test for dimensional differences in autism traits by applying a population-based approach, in order to obtain samples reflecting the epidemiological prevalence of autism, alexithymia, or other psychological diagnoses (Abu-Akel et al., 2019). Second, this study was cross-sectional and therefore limited in allowing inferences on development. Future studies in adolescents should use a longitudinal design in order to better understand the origin and development of relationships between self-evaluation, underlying neural mechanisms, during childhood and adolescence. Third, unlike the participants with autism, participants without autism had no (additional) mental struggles and did not use any medication. However, this strengthens our null results regarding group differences since overlapping additional issues in both groups would make it even more difficult to strictly differentiate between groups. Fourth, due to practical limitations, this study was limited to the inclusion of adolescent males, even though the male-to-female ratio among children and adolescents with ASC is about 3 to 1 (Loomes et al., 2017). It should be kept in mind that results may be different for autistic adolescent females, especially since adolescent girls are generally lower on self-esteem (Chung et al., 2014; Robins & Trzesniewski, 2005), and self-positivity may differ between the sexes depending on the domain (Cole et al., 2001; Gentile et al., 2009; Marsh & Ayotte, 2003). Therefore, future studies should aim to also include adolescent females. Fifth, although autistic traits as measured with the AQ-short significantly differed between groups, there was a participant in the autism group who scored very low (3), and some participants in the non-autistic group scored rather high (up to 22). Future studies may aim to use an additional measure to get an indication of autism traits. Sixth, future studies may consider investigating group differences using Bayesian inference to calculate the probability of the null hypothesis being true.

### Conclusion

This study did not observe group differences between autistic and non-autistic adolescents, but confirmed that self-concept positivity (specifically physical appearance and prosocial) and self-esteem were lower in adolescent males with more autism traits. These findings fit with recent theories showing that a dimensional approach is more suitable in mental health research (Conway et al., 2019) as autism traits may also be present along a continuum in the general non-diagnosed population. In addition, evaluating self-traits largely relies on the same neural activation in adolescents with and without autism (Cage et al., 2016), suggesting that adolescents make use of the same underlying neural processes when evaluating self-traits.

Last, this study confirmed that alexithymia is predominantly related to the social-emotional facets of evaluating the self (Bernhardt et al., 2014; Cook et al., 2013; Milosavljevic et al., 2016; Moriguchi et al., 2006). Several social-emotional problems traditionally associated with ASCs such as empathic deficits, emotion recognition difficulties, mentalizing impairments, and less interoceptive awareness have been shown to be related to alexithymia rather than autism (i.e. alexithymia hypothesis; Cook et al., 2013; Lombardo et al., 2007; Milosavljevic et al., 2016; Moriguchi et al., 2006; Shah et al., 2016). Internalizing problems often co-occurring with ASCs such as depression and anxiety may be explained by alexithymia as well (Bloch et al., 2021; Milosavljevic et al., 2016). Since negative self-concept and low self-esteem are risk factors for developing internalizing problems that increase during adolescence (Giedd et al., 2008; Kessler et al., 2005; Van Tuijl et al., 2014) and that often co-occur with ASC (Simonoff et al., 2008), future studies should examine negative self-views in adolescents with and without autism in more detail.

Together, this study informs future studies by providing a comprehensive view of adolescent self-concept and self-esteem in relation to autism and alexithymia traits, and it applies to clinical practice where it may be important to take both autism and alexithymia into account in treatments of clients with low self-esteem.

## Supplemental Material

sj-docx-1-aut-10.1177_13623613241232860 – Supplemental material for The role of autism and alexithymia traits in behavioral and neural indicators of self-concept and self-esteem in adolescence Supplemental material, sj-docx-1-aut-10.1177_13623613241232860 for The role of autism and alexithymia traits in behavioral and neural indicators of self-concept and self-esteem in adolescence by Renske van der Cruijsen, Sander Begeer and Eveline A Crone in Autism
