# Supplementary material for: The role of autism and alexithymia traits in behavioral and neural indicators of self-concept and self-esteem in adolescence
Source: Autism. 2024 Feb 27;28(9):2346–61. doi: 10.1177/13623613241232860 (PMC11403929; doi:10.1177/13623613241232860)
Supplement: sj-docx-1-aut-10.1177_13623613241232860 – Supplemental material for The role of autism and alexithymia traits in behavioral and neural indicators of self-concept and self-esteem in adolescence [file sj-docx-1-aut-10.1177_13623613241232860.docx]

**Supplement**

**fMRI data acquisition**

A Philips 3T MRI scanner with a standard whole-head coil was used to acquire MRI scans. Functional scans were acquired in three runs using T2*-weighted echo-planar imaging (EPI) sequence (TR=2200msec, TE=30msec, sequential acquisition, 37slices of 2.75mm, FOV=220x220x111.65mm). The first two volumes of each run were discarded to account for T1 saturation. A high-resolution 3D T1-FFE scan for anatomical reference was obtained (TR-shortest msec, TE=4.6msec, 140slices, voxel size=.875mm, FOV=224x178.5x168mm). Trait sentences were projected on a screen behind the scanner and could be seen by the participant via a mirror attached to the head coil. Head movement was restricted by placing foam inserts inside the coil.

**Supplementary Results**

**Hierarchical regression analyses for the ASD group only**

To provide additional insight into relationships of autism and alexithymia traits with the behavioral and neural measures of self-concept, all hierarchical regression analyses were repeated within the ASD group only. Results are briefly summarized below.

***Behavioral results***

**Self-concept positivity.** In contrast to analyses in the complete group of participants, autism traits were not related to self-concept positivity (all *p*-values > .050). Here, adding alexithymia improved model fit for self-concept positivity in the academic domain (*F*_change_(2, 28)=4.025, *R*²_change_=.205, *p*_change_=.029; Difficulty identifying feelings: *β*=-.583, *t*(28)=-2.72, *p*=.011).

**Self-esteem.** In contrast to analyses in the complete group of participants, autism traits were not related to self-esteem (*β*=-.229, *t*(30)=-1.231, *p*=.228). Similar to analyses in the complete group of participants, the model improved after adding alexithymia. In this model, difficulty identifying feelings (DIF) was negatively related to self-esteem (*F*_change_(2, 28)=5.223, *R*²_change_=.250, *p*_change_=.012; AQ: *β*=-.399, *t*(30)=-2.297, *p*=.029).

**Perspective similarity.** As in the main analyses, item-item agreement in the prosocial domain was negatively related to autism traits (*F*_change_(1, 30)=5.110, *R*²_change_=.140, *p*_change_=.031; Difficulty identifying feelings: *β*=-.397, *t*(28)=-2.261, *p*=.031). All other *p*-values >.070.

***Neural results***

**Self-related brain activation: Self > Control.** In line with analyses in the complete group of participants, autism traits were never related to neural activation (all *p*-values > .100), and alexithymia traits were not related to neural activation in left and right TPJ (all *p*-values >.150). In contrast to the main analyses, mPFC activation was not related to alexithymia traits (all *p*-values > .249).

**Perspective similarity brain activation: Reflected > Direct.** Similar to the main analyses, both mPFC and left TPJ activation for reflected > direct self-concept (in general and for all domains separately) was not related to autism or alexithymia traits (all *p*-values > .087).

As in the analyses in the complete group of participants, autism traits were not related to right TPJ activation in the contrast Reflected > Direct (all *p*-values > .506). Adding alexithymia DIF and DDF in step 3 of the regression significantly improved the model across all domains, and for the academic and physical domains specifically (across domains: *F*_change_(2, 28)=3.872, *R*²_change_=.169, *p*_change_=.033; academic: *F*_change_(2, 28)=3.589, *R*²_change_=.169, *p*_change_=.041; physical: *F*_change_(2, 28)=3.825, *R*²_change_=.177, *p*_change_=.034). Here, difficulty identifying feelings positively related to the difference in right TPJ activation between reflected and direct self-evaluations (across domains: *β*=.514, *t*(28)=2.592, *p*=.015; academic: *β*=.512, *t*(28)=2.492, *p*=.019; physical: *β*=.463, *t*(28)=2.271, *p*=.031). Alexithymia did not improve the model for the prosocial domain (*p*=.224).

Table S1. Statistics for the hierarchical regression models - behavior

|  | **R^2^change** | **Fchange** | ***p* change** | ***β*** | ***t*** | ***p*** |
| --- | --- | --- | --- | --- | --- | --- |
| **Self-concept positivity** | |  |  |  |  |  |
| **Academic Self**  ***Step 1*** | |  |  |  |  |  |
| Medication |  |  |  | -.052 | -.424 | .673 |
| IQ |  |  |  | .153 | 1.252 | .215 |
| Age |  |  |  | -.081 | -.665 | .508 |
| ***Step 2*** | .003 | .203 | .654 |  |  |  |
| AQ |  |  |  | -.059 | -.450 | .654 |
| ***Step 3*** | .050 | 1.696 | .192 |  |  |  |
| DIF |  |  |  | -.253 | -1.598 | .115 |
| DDF |  |  |  | .022 | .135 | .893 |
|  |  |  |  |  |  |  |
| **Physical Self**  ***Step 1*** |  |  |  |  |  |  |
| Medication |  |  |  | -.233 | -1.976 | .052 |
| IQ |  |  |  | -.089 | -.750 | .456 |
| Age |  |  |  | .204 | 1.730 | .088 |
| ***Step 2*** | .134 | 11.235 | .001 |  |  |  |
| AQ |  |  |  | -.392 | -3.352 | **.001** |
| ***Step 3*** | .040 | 1.695 | .192 |  |  |  |
| DIF |  |  |  | -.166 | -1.177 | .244 |
| DDF |  |  |  | -.076 | -.515 | .608 |
|  |  |  |  |  |  |  |
| **Prosocial Self**  ***Step 1*** |  |  |  |  |  |  |
| Medication |  |  |  | -.155 | -1.270 | .209 |
| IQ |  |  |  | .075 | .618 | .539 |
| Age |  |  |  | .103 | .849 | .399 |
| ***Step 2*** | .183 | 15.117 | <.001 |  |  |  |
| AQ |  |  |  | -.459 | -3.888 | **<.001** |
| ***Step 3*** | .057 | 2.472 | .093 |  |  |  |
| DIF |  |  |  | .251 | 1.791 | .078 |
| DDF |  |  |  | -.310 | -2.094 | .040^+^ |
|  |  |  |  |  |  |  |
| **Self-esteem** |  |  |  |  |  |  |
| **Self-esteem**  ***Step 1*** |  |  |  |  |  |  |
| Medication |  |  |  | .115 | .938 | .352 |
| IQ |  |  |  | -.062 | -.502 | .617 |
| Age |  |  |  | .044 | .358 | .722 |
| ***Step 2*** | .103 | 7.505 | .008 |  |  |  |
| AQ |  |  |  | -.343 | -2.739 | **.008*** |
| ***Step 3*** | .180 | 8.027 | <.001 |  |  |  |
| DIF |  |  |  | -.495 | -3.584 | **<.001** |
| DDF |  |  |  | .076 | .520 | .605 |
|  |  |  |  |  |  |  |
|  |  |  |  |  |  |  |
| **Item-item agreement** | |  |  |  |  |  |
| **Academic agreement**  ***Step 1*** | |  |  |  |  |  |
| Medication |  |  |  | .052 | .416 | .679 |
| IQ |  |  |  | .023 | .182 | .856 |
| Age |  |  |  | -.007 | -.054 | .957 |
| ***Step 2*** | .011 | .701 | .406 |  |  |  |
| AQ |  |  |  | -.111 | -.837 | .406 |
| ***Step 3*** | .065 | 2.179 | .122 |  |  |  |
| DIF |  |  |  | -.332 | -2.087 | .041^+^ |
| DDF |  |  |  | .203 | 1.211 | .230 |
|  |  |  |  |  |  |  |
| **Physical agreement**  ***Step 1*** | |  |  |  |  |  |
| Medication |  |  |  | .070 | .585 | .561 |
| IQ |  |  |  | .127 | 1.061 | .292 |
| Age |  |  |  | .254 | 2.132 | .037^+^ |
| ***Step 2*** | .039 | 2.823 | .098 |  |  |  |
| AQ |  |  |  | -.211 | -1.680 | .098 |
| ***Step 3*** | .001 | .042 | .959 |  |  |  |
| DIF |  |  |  | -.045 | -.288 | .774 |
| DDF |  |  |  | .023 | .142 | .887 |
|  |  |  |  |  |  |  |
| **Prosocial agreement**  ***Step 1*** | |  |  |  |  |  |
| Medication |  |  |  | .074 | .604 | .548 |
| IQ |  |  |  | .008 | .064 | .949 |
| Age |  |  |  | .125 | 1.015 | .314 |
| ***Step 2*** | .139 | 10.567 | .002 |  |  |  |
| AQ |  |  |  | -.399 | -3.251 | **.002** |
| ***Step 3*** | .022 | .844 | .435 |  |  |  |
| DIF |  |  |  | .128 | .852 | .397 |
| DDF |  |  |  | .053 | .336 | .738 |

^+^did not survive Bonferroni correction

Table S2. Statistics for the hierarchical regression models – MRI: Self vs Control

|  | **R^2^change** | **Fchange** | ***p* change** | ***β*** | ***t*** | ***p*** |
| --- | --- | --- | --- | --- | --- | --- |
| **mPFC Self > Control** | |  |  |  |  |  |
|  | |  |  |  |  |  |
| **mPFC General**  ***Step 1*** |  |  |  |  |  |  |
| Medication |  |  |  | .006 | .053 | .958 |
| IQ |  |  |  | .053 | .431 | .668 |
| Age |  |  |  | .171 | 1.397 | .167 |
| ***Step 2*** | .001 | .068 | .796 |  |  |  |
| AQ |  |  |  | -.034 | -.260 | .796 |
| ***Step 3*** | .159 | 6.069 | .004 |  |  |  |
| DIF |  |  |  | -.475 | -3.185 | **.002** |
| DDF |  |  |  | .463 | 2.952 | **.004** |
|  |  |  |  |  |  |  |
| **mPFC Academic**  ***Step 1*** | |  |  |  |  |  |
| Medication |  |  |  | .009 | .075 | .940 |
| IQ |  |  |  | .098 | .804 | .424 |
| Age |  |  |  | .216 | 1.78 | .080 |
| ***Step 2*** | .000 | .002 | .963 |  |  |  |
| AQ |  |  |  | .006 | .047 | .963 |
| ***Step 3*** | .128 | 4.864 | .011 |  |  |  |
| DIF |  |  |  | -.427 | -2.848 | **.006*** |
| DDF |  |  |  | .418 | 2.647 | **.010*** |
|  |  |  |  |  |  |  |
| **mPFC Physical**  ***Step 1*** | |  |  |  |  |  |
| Medication |  |  |  | -.008 | -.069 | .945 |
| IQ |  |  |  | .025 | .203 | .840 |
| Age |  |  |  | .176 | 1.437 | .155 |
| ***Step 2*** | .001 | .055 | .815 |  |  |  |
| AQ |  |  |  | -.031 | -.235 | .815 |
| ***Step 3*** | .120 | 4.381 | .017 |  |  |  |
| DIF |  |  |  | -.426 | -2.792 | **.007*** |
| DDF |  |  |  | .382 | 2.377 | .021^+^ |
|  |  |  |  |  |  |  |
| **mPFC Prosocial**  ***Step 1*** | |  |  |  |  |  |
| Medication |  |  |  | .018 | .145 | .885 |
| IQ |  |  |  | .033 | .268 | .790 |
| Age |  |  |  | .105 | .850 | .398 |
| ***Step 2*** | .004 | .278 | .600 |  |  |  |
| AQ |  |  |  | -.070 | -.527 | .600 |
| ***Step 3*** | .192 | 7.537 | .001 |  |  |  |
| DIF |  |  |  | -.508 | -3.451 | **.001** |
| DDF |  |  |  | .528 | 3.405 | **.001** |
|  |  |  |  |  |  |  |
|  |  |  |  |  |  |  |
|  |  |  |  |  |  |  |
|  |  |  |  |  |  |  |
| **Left TPJ Self > Control** | |  |  |  |  |  |
|  |  |  |  |  |  |  |
| **Left TPJ General**  ***Step 1*** | | |  |  |  |  |
| Medication |  |  |  | .104 | .846 | .401 |
| IQ |  |  |  | -.051 | -.409 | .684 |
| Age |  |  |  | -.028 | -.226 | .822 |
| ***Step 2*** | .054 | 3.680 | .060 |  |  |  |
| AQ |  |  |  | .248 | 1.918 | .060 |
| ***Step 3*** | .009 | .311 | .734 |  |  |  |
| DIF |  |  |  | .087 | .548 | .586 |
| DDF |  |  |  | .028 | .165 | .870 |
|  |  |  |  |  |  |  |
| **Left TPJ Academic**  ***Step 1*** | | |  |  |  |  |
| Medication |  |  |  | .076 | .615 | .541 |
| IQ |  |  |  | -.071 | -.570 | .570 |
| Age |  |  |  | -.018 | -.148 | .883 |
| ***Step 2*** | .080 | 5.623 | .021 |  |  |  |
| AQ |  |  |  | .303 | 2.371 | .021^+^ |
| ***Step 3*** | <.001 | .016 | .984 |  |  |  |
| DIF |  |  |  | .016 | .102 | .919 |
| DDF |  |  |  | .011 | .064 | .949 |
|  |  |  |  |  |  |  |
| **Left TPJ Physical**  ***Step 1*** | | |  |  |  |  |
| Medication |  |  |  | .105 | .857 | .395 |
| IQ |  |  |  | -.080 | -.646 | .520 |
| Age |  |  |  | .012 | .096 | .924 |
| ***Step 2*** | .043 | 2.964 | .090 |  |  |  |
| AQ |  |  |  | .223 | 1.722 | .090 |
| ***Step 3*** | .012 | .396 | .674 |  |  |  |
| DIF |  |  |  | .130 | .813 | .419 |
| DDF |  |  |  | -.026 | -.155 | .878 |
|  |  |  |  |  |  |  |
| **Left TPJ Prosocial**  ***Step 1*** | |  |  |  |  |  |
| Medication |  |  |  | .116 | .945 | .348 |
| IQ |  |  |  | .007 | .057 | .955 |
| Age |  |  |  | -.076 | -.616 | .540 |
| ***Step 2*** | .031 | 2.062 | .156 |  |  |  |
| AQ |  |  |  | .187 | 1.436 | .156 |
| ***Step 3*** | .024 | .817 | .446 |  |  |  |
| DIF |  |  |  | .099 | .620 | .538 |
| DDF |  |  |  | .097 | .580 | .564 |
|  |  |  |  |  |  |  |
|  |  |  |  |  |  |  |
|  |  |  |  |  |  |  |
| **Right TPJ Self > Control** | |  |  |  |  |  |
|  |  |  |  |  |  |  |
| **Right TPJ General**  ***Step 1*** | |  |  |  |  |  |
| Medication |  |  |  | .052 | .421 | .675 |
| IQ |  |  |  | .060 | .489 | .626 |
| Age |  |  |  | -.106 | -.857 | .394 |
| ***Step 2*** | .007 | .452 | .504 |  |  |  |
| AQ |  |  |  | .089 | .672 | .504 |
| ***Step 3*** | .003 | .085 | .919 |  |  |  |
| DIF |  |  |  | .048 | .295 | .769 |
| DDF |  |  |  | .013 | .073 | .942 |
|  |  |  |  |  |  |  |
| **Right TPJ Academic**  ***Step 1*** | |  |  |  |  |  |
| Medication |  |  |  | .037 | .299 | .766 |
| IQ |  |  |  | .072 | .581 | .564 |
| Age |  |  |  | -.114 | -.928 | .357 |
| ***Step 2*** | .012 | .793 | .377 |  |  |  |
| AQ |  |  |  | .117 | .890 | .377 |
| ***Step 3*** | <.001 | .013 | .987 |  |  |  |
| DIF |  |  |  | -.010 | -.063 | .950 |
| DDF |  |  |  | .027 | .160 | .874 |
|  |  |  |  |  |  |  |
| **Right TPJ Physical**  ***Step 1*** | |  |  |  |  |  |
| Medication |  |  |  | .081 | .659 | .512 |
| IQ |  |  |  | .031 | .247 | .806 |
| Age |  |  |  | -.066 | -.537 | .593 |
| ***Step 2*** | .012 | .755 | .388 |  |  |  |
| AQ |  |  |  | .115 | .869 | .388 |
| ***Step 3*** | .002 | .061 | .940 |  |  |  |
| DIF |  |  |  | .056 | .343 | .733 |
| DDF |  |  |  | -.043 | -.251 | .802 |
|  |  |  |  |  |  |  |
| **Right TPJ Prosocial**  ***Step 1*** | |  |  |  |  |  |
| Medication |  |  |  | .033 | .267 | .791 |
| IQ |  |  |  | .071 | .578 | .565 |
| Age |  |  |  | -.123 | -1.000 | .321 |
| ***Step 2*** | .001 | .043 | .836 |  |  |  |
| AQ |  |  |  | .028 | .208 | .836 |
| ***Step 3*** | .013 | .422 | .658 |  |  |  |
| DIF |  |  |  | .091 | .559 | .578 |
| DDF |  |  |  | .050 | .291 | .772 |

^+^did not survive Bonferroni correction * Did not survive additional Bonferroni correction

Table S3. Statistics for the hierarchical regression models – MRI: Reflected vs Direct

|  | **R^2^change** | **Fchange** | | ***p* change** | | ***β*** | | ***t*** | | ***p*** | |  |
| --- | --- | --- | --- | --- | --- | --- | --- | --- | --- | --- | --- | --- |
| **mPFC Reflected > Direct** | |  | |  | |  | |  | |  | |  |
|  | |  | |  | |  | |  | |  | |  |
| **mPFC General**  ***Step 1*** |  |  | |  | |  | |  | |  | |  |
| Medication |  |  | |  | | -.178 | | -1.494 | | .140 | |  |
| IQ |  |  | |  | | -.236 | | -1.979 | | .052 | |  |
| Age |  |  | |  | | .030 | | .255 | | .800 | |  |
| ***Step 2*** | .007 | .482 | | .490 | |  | |  | |  | |  |
| AQ |  |  | |  | | -.089 | | -.694 | | .490 | |  |
| ***Step 3*** | .065 | 2.385 | | .101 | |  | |  | |  | |  |
| DIF |  |  | |  | | .168 | | 1.105 | | 273 | |  |
| DDF |  |  | |  | | .151 | | .943 | | .349 | |  |
|  |  |  | |  | |  | |  | |  | |  |
| **mPFC Academic**  ***Step 1*** | |  | |  | |  | |  | |  | |  |
| Medication |  |  | |  | | -.152 | | -1.261 | | .212 | |  |
| IQ |  |  | |  | | -.200 | | -1.654 | | .103 | |  |
| Age |  |  | |  | | .004 | | .032 | | .974 | |  |
| ***Step 2*** | .003 | .193 | | .662 | |  | |  | |  | |  |
| AQ |  |  | |  | | .057 | | .439 | | .662 | |  |
| ***Step 3*** | .077 | 2.781 | | .070 | |  | |  | |  | |  |
| DIF |  |  | |  | | .219 | | 1.425 | | .159 | |  |
| DDF |  |  | |  | | .123 | | .761 | | .450 | |  |
|  |  |  | |  | |  | |  | |  | |  |
| **mPFC Physical**  ***Step 1*** | |  | |  | |  | |  | |  | |  |
| Medication |  |  | |  | | -.112 | | -.923 | | .360 | |  |
| IQ |  |  | |  | | -.133 | | 1.095 | | .278 | |  |
| Age |  |  | |  | | .135 | | 1.109 | | .271 | |  |
| ***Step 2*** | .026 | 1.780 | | .187 | |  | |  | |  | |  |
| AQ |  |  | |  | | -.172 | | -1.334 | | .187 | |  |
| ***Step 3*** | .052 | 1.860 | | .164 | |  | |  | |  | |  |
| DIF |  |  | |  | | .094 | | .610 | | .544 | |  |
| DDF |  |  | |  | | .191 | | 1.175 | | .244 | |  |
|  |  |  | |  | |  | |  | |  | |  |
| **mPFC Prosocial**  ***Step 1*** | |  | |  | |  | |  | |  | |  |
| Medication |  |  | |  | | -.140 | | -1.177 | | .243 | |  |
| IQ |  |  | |  | | -.269 | | -2.257 | | .027^+^ | |  |
| Age |  |  | |  | | -.019 | | -.156 | | .877 | |  |
| ***Step 2*** | .016 | 1.176 | | .282 | |  | |  | |  | |  |
| AQ |  |  | |  | | -.138 | | -1.084 | | .282 | |  |
| ***Step 3*** | .016 | .559 | | .575 | |  | |  | |  | |  |
| DIF |  |  | |  | | .161 | | 1.036 | | .304 | |  |
| DDF |  |  | |  | | -.066 | | -.404 | | .688 | |  |
|  |  |  | |  | |  | |  | |  | |  |
|  |  |  | |  | |  | |  | |  | |  |
|  |  |  | |  | |  | |  | |  | |  |
|  |  |  | |  | |  | |  | |  | |  |
| **Left TPJ Reflected > Direct** | |  | |  | |  | |  | |  | |  |
|  |  |  | |  | |  | |  | |  | |  |
| **Left TPJ General**  ***Step 1*** | | | |  | |  | |  | |  | |  |
| Medication |  |  | |  | | -.044 | | -.353 | | .725 | |  |
| IQ |  |  | |  | | .006 | | .050 | | .960 | |  |
| Age |  |  | |  | | .040 | | .320 | | .750 | |  |
| ***Step 2*** | .033 | 2.162 | | .146 | |  | |  | |  | |  |
| AQ |  |  | |  | | -.193 | | -1.471 | | .146 | |  |
| ***Step 3*** | .041 | 1.367 | | .262 | |  | |  | |  | |  |
| DIF |  |  | |  | | .263 | | 1.652 | | .104 | |  |
| DDF |  |  | |  | | -.165 | | -.983 | | .330 | |  |
|  |  |  | |  | |  | |  | |  | |  |
| **Left TPJ Academic**  ***Step 1*** | | | |  | |  | |  | |  | |  |
| Medication |  |  | |  | | -.116 | | -.940 | | .351 | |  |
| IQ |  |  | |  | | -.061 | | -.496 | | .621 | |  |
| Age |  |  | |  | | .008 | | .066 | | .948 | |  |
| ***Step 2*** | .003 | .205 | | .653 | |  | |  | |  | |  |
| AQ |  |  | |  | | -.060 | | -.452 | | .653 | |  |
| ***Step 3*** | .076 | 2.614 | | .081 | |  | |  | |  | |  |
| DIF |  |  | |  | | .356 | | 2.264 | | .027^+^ | |  |
| DDF |  |  | |  | | -.166 | | -.999 | | .322 | |  |
|  |  |  | |  | |  | |  | |  | |  |
| **Left TPJ Physical**  ***Step 1*** | | | |  | |  | |  | |  | |  |
| Medication |  |  | |  | | .004 | | .029 | | .977 | |  |
| IQ |  |  | |  | | .012 | | .100 | | .921 | |  |
| Age |  |  | |  | | .097 | | .784 | | .436 | |  |
| ***Step 2*** | .018 | 1.179 | | .282 | |  | |  | |  | |  |
| AQ |  |  | |  | | -.143 | | -1.086 | | .282 | |  |
| ***Step 3*** | .024 | .778 | | .464 | |  | |  | |  | |  |
| DIF |  |  | |  | | .196 | | 1.214 | | .230 | |  |
| DDF |  |  | |  | | -.075 | | -.439 | | .662 | |  |
|  |  |  | |  | |  | |  | |  | |  |
| **Left TPJ Prosocial**  ***Step 1*** | |  | |  | |  | |  | |  | |  |
| Medication |  |  | |  | | -.008 | | -.066 | | .947 | |  |
| IQ |  |  | |  | | .061 | | .491 | | .625 | |  |
| Age |  |  | |  | | -.018 | | -.141 | | .888 | |  |
| ***Step 2*** | .070 | 4.831 | | .032 | |  | |  | |  | |  |
| AQ |  |  | |  | | -.283 | | -2.198 | | .032^+^ | |  |
| ***Step 3*** | .019 | .644 | | .528 | |  | |  | |  | |  |
| DIF |  |  | |  | | .119 | | .753 | | .454 | |  |
| DDF |  |  | |  | | -.187 | | -1.125 | | .265 | |  |
|  |  |  | |  | |  | |  | |  | |  |
|  |  |  | |  | |  | |  | |  | |  |
|  |  |  | |  | |  | |  | |  | |  |
|  |  |  | |  | |  | |  | |  | |  |
|  |  |  | |  | |  | |  | |  | |  |
|  |  |  | |  | |  | |  | |  | |  |
|  |  |  | |  | |  | |  | |  | |  |
| **Right TPJ Reflected > Direct** | | |  | |  | |  | |  | |  | |
|  |  |  | |  | |  | |  | |  | |  |
| **Right TPJ General**  ***Step 1*** | |  | |  | |  | |  | |  | |  |
| Medication |  |  | |  | | -.175 | | -1.475 | | .145 | |  |
| IQ |  |  | |  | | -.260 | | -2.188 | | .032^+^ | |  |
| Age |  |  | |  | | .025 | | .209 | | .835 | |  |
| ***Step 2*** | .006 | .422 | | .518 | |  | |  | |  | |  |
| AQ |  |  | |  | | -.083 | | -.650 | | .518 | |  |
| ***Step 3*** | .120 | 4.786 | | .012 | |  | |  | |  | |  |
| DIF |  |  | |  | | .451 | | 3.085 | | **.003** | |  |
| DDF |  |  | |  | | -.236 | | -1.532 | | .131 | |  |
|  |  |  | |  | |  | |  | |  | |  |
| **Right TPJ Academic**  ***Step 1*** | |  | |  | |  | |  | |  | |  |
| Medication |  |  | |  | | -.150 | | -1.235 | | .221 | |  |
| IQ |  |  | |  | | -.163 | | -1.338 | | .186 | |  |
| Age |  |  | |  | | -.082 | | -.679 | | .500 | |  |
| ***Step 2*** | <.001 | .001 | | .974 | |  | |  | |  | |  |
| AQ |  |  | |  | | .004 | | .033 | | .974 | |  |
| ***Step 3*** | .132 | 4.999 | | .010 | |  | |  | |  | |  |
| DIF |  |  | |  | | .463 | | 3.090 | | **.003** | |  |
| DDF |  |  | |  | | -.185 | | -1.173 | | .245 | |  |
|  |  |  | |  | |  | |  | |  | |  |
| **Right TPJ Physical**  ***Step 1*** | |  | |  | |  | |  | |  | |  |
| Medication |  |  | |  | | -.179 | | -1.528 | | .131 | |  |
| IQ |  |  | |  | | -.244 | | -2.077 | | .042^+^ | |  |
| Age |  |  | |  | | .138 | | 1.175 | | .244 | |  |
| ***Step 2*** | <.001 | .005 | | .944 | |  | |  | |  | |  |
| AQ |  |  | |  | | -.009 | | -.070 | | .944 | |  |
| ***Step 3*** | .099 | 3.876 | | .026 | |  | |  | |  | |  |
| DIF |  |  | |  | | .389 | | 2.643 | | **.010*** | |  |
| DDF |  |  | |  | | -.117 | | -.752 | | .455 | |  |
|  |  |  | |  | |  | |  | |  | |  |
| **Right TPJ Prosocial**  ***Step 1*** | |  | |  | |  | |  | |  | |  |
| Medication |  |  | |  | | -.107 | | -.895 | | .374 | |  |
| IQ |  |  | |  | | -.250 | | -2.081 | | .041^+^ | |  |
| Age |  |  | |  | | .001 | | .012 | | .990 | |  |
| ***Step 2*** | .041 | 2.989 | | .089 | |  | |  | |  | |  |
| AQ |  |  | |  | | -.218 | | -1.729 | | .089 | |  |
| ***Step 3*** | .061 | 2.276 | | .111 | |  | |  | |  | |  |
| DIF |  |  | |  | | .277 | | 1.840 | | .071 | |  |
| DDF |  |  | |  | | -.305 | | -1.925 | | .059 | |  |

^+^did not survive Bonferroni correction * Did not survive additional Bonferroni correction
